# Gendered health systems: evidence from low- and middle-income countries

**DOI:** 10.1186/s12961-018-0338-5

**Published:** 2018-07-06

**Authors:** Rosemary Morgan, Richard Mangwi Ayiasi, Debjani Barman, Stephen Buzuzi, Charles Ssemugabo, Nkoli Ezumah, Asha S. George, Kate Hawkins, Xiaoning Hao, Rebecca King, Tianyang Liu, Sassy Molyneux, Kelly W. Muraya, David Musoke, Tumaini Nyamhanga, Bandeth Ros, Kassimu Tani, Sally Theobald, Sreytouch Vong, Linda Waldman

**Affiliations:** 1 Department of International Health, Johns Hopkins Bloomberg School of Public Health, 615 N. Wolfe Street, Baltimore, MD 21205 United States of America; 2 Makerere University, School of Public Health, College of Health Sciences, P.O. Box 7072, Kampala, Uganda; 3 IIHMR University, 1 Prabhu Dayal Marg, Near Sanganer Airport, Jaipur, 302029 India; 4 Biomedical Research and Training Institute, 10 Seagrave Road, Avondale, Harare, Zimbabwe; 5 Department of Disease Control and Environmental Health, School of Public Health, Makerere University College of Health Science, P.O. Box 7072, Kampala, Uganda; 6 Health Policy Research Group, College of Medicine, University of Nigeria, Enugu Campus, Enugu, Nigeria; 7 Department of Sociology/Anthropology, University of Nigeria, Nsukka, Nigeria; 8 School of Public Health, University of Western Cape, Private Bag x17, Bellville, Cape Town, 7535 South Africa; 9 Pamoja Communications Ltd., 81 Ewhurst Road, Brighton, BN2 4AL United Kingdom; 10 China National Health Development Research Center, NO.38 Xueyuan Road, Haidian District, Beijing, China; 11 Nuffield Centre for International Health and Development, Leeds Institute of Health Sciences, University of Leeds, Leeds, LS2 9NL United Kingdom; 12 Kenya Medical Research Institute (KEMRI) - Wellcome Trust Research Programme, PO Box 43640-00100, Nairobi, Kenya; 13 Nuffield Department of Medicine, Oxford University, Oxford, United Kingdom; 14 Department of Development Studies, Muhimbili University of Health and Allied Sciences, P.O. Box 65454, Dar es Salaam, Tanzania; 15 ReBUILD and RinGs Consortia, Phnom Penh, Cambodia; 16 Ifakara Health Institute, P.O. Box 78373, Dar es Salaam, Tanzania; 17 Social Science and International Health, Liverpool School of Tropical Medicine, Pembroke Pl, Liverpool, L3 5QA United Kingdom; 18 Institute of Development Studies, Library Road, Brighton, BN1 9RE United Kingdom

**Keywords:** Gender, Gender analysis, Intersectionality, Health systems research, Financing, Service delivery, Human resources for health, Governance

## Abstract

**Background:**

Gender is often neglected in health systems, yet health systems are not gender neutral. Within health systems research, gender analysis seeks to understand how gender power relations create inequities in access to resources, the distribution of labour and roles, social norms and values, and decision-making. This paper synthesises findings from nine studies focusing on four health systems domains, namely human resources, service delivery, governance and financing. It provides examples of how a gendered and/or intersectional gender approach can be applied by researchers in a range of low- and middle-income settings (Cambodia, Zimbabwe, Uganda, India, China, Nigeria and Tanzania) to issues across the health system and demonstrates that these types of analysis can uncover new and novel ways of viewing seemingly intractable problems.

**Methods:**

The research used a combination of mixed, quantitative, qualitative and participatory methods, demonstrating the applicability of diverse research methods for gender and intersectional analysis. Within each study, the researchers adapted and applied a variety of gender and intersectional tools to assist with data collection and analysis, including different gender frameworks. Some researchers used participatory tools, such as photovoice and life histories, to prompt deeper and more personal reflections on gender norms from respondents, whereas others used conventional qualitative methods (in-depth interviews, focus group discussion). Findings from across the studies were reviewed and key themes were extracted and summarised.

**Results:**

Five core themes that cut across the different projects were identified and are reported in this paper as follows: the intersection of gender with other social stratifiers; the importance of male involvement; the influence of gendered social norms on health system structures and processes; reliance on (often female) unpaid carers within the health system; and the role of gender within policy and practice. These themes indicate the relevance of and need for gender analysis within health systems research.

**Conclusion:**

The implications of the diverse examples of gender and health systems research highlighted indicate that policy-makers, health practitioners and others interested in enhancing health system research and delivery have solid grounds to advance their enquiry and that one-size-fits-all heath interventions that ignore gender and intersectionality dimensions require caution. It is essential that we build upon these insights in our efforts and commitment to move towards greater equity both locally and globally.

## Background

Gender is often neglected in health systems, yet health systems are not gender neutral. Gender is a key social stratifier, affecting health system needs, experiences and outcomes at all levels [1–3]. Within health systems research, gender analysis seeks to understand how gender power relations create inequities in access to resources, the distribution of labour and roles, social norms and values, and decision-making [1, 3–5]. An intersectional gender analysis [6], which aims to promote researcher and activist engagement to bring about positive transformation in the structures and institutions of power, explores how gender intersects with other determinants of social stratification, such as race, class, age, (dis)ability, education, etc., to create different experiences of privilege and/or marginalisation within the health system [7]. Intersectionality offers an analysis that augments our understanding of gender, and how gender and other social stratifiers are mutually constituted and intersect in dynamic and interactive ways [8, 9].

The Research in Gender and Ethics (RinGs): Building Stronger Health Systems Consortium [10] provided funding to researchers in Africa and Asia to explore the role of gender across health systems. Research focused on four health systems domains, including human resources, service delivery, governance and financing (Table 1). This paper synthesises findings from nine of these studies, including the methodologies used to incorporate gender analysis into health systems research. It provides examples of how a gendered and/or intersectional gender approach can be applied by researchers in a range of low- and middle-income settings to issues across the health system and demonstrates that these types of analysis can uncover new and novel ways of viewing seemingly intractable problems.

**Table 1:** Overview of methodology, methods and participants included in country studies

Title	Location	Study design and methods	Data sources	Participants	Analysis approach
Human Resources					
Women and Leadership within the Cambodia Health Sector	Battambang province, Cambodia	Qualitative, life history interviews	In-depth interviews (*N* = 20)	Health managers (14 F, 6 M)	Thematic analysis
Gender Mainstreaming in the Posting and Deployment of Health Workers in Zimbabwe	Midlands province, Zimbabwe	Cross-sectional mixed-methods design; Life history interviews	Policy and documentreview	National Gender Policy, Public Service Regulations, Manpower Development Plan	Thematic analysis using a framework approach
			In-depth interviews (*N* = 30)	Health workers (nurses, midwives and environmental health technicians), *N* = 19 (8 M, 11 F) Human resource officers/managers *N* = 11 (6 M, 5F)	
Exploring Gendered Experiences of Community Health Workers Using Photovoice in Rural Wakiso District, Uganda	Wakiso district, Uganda	Qualitative community-based participatory approach using photovoice	Discussions during meetings around photographs	Community health workers, *N* = 10 (5 F, 5 M)	Conventional content analysis using Atlas ti version 6.0.15
Service Delivery					
Why are Maternal Health Outcomes Worse for Migrant Women in Masindi, Uganda?	Nyantonzi Parish, Masindi district, western Uganda	Qualitative	FGDs (*N* = 5)	Migrant women who have recently given birth Migrant pregnant women Spouses of migrant women whose wives were pregnant Spouses of migrant women whose wives recently gave birth	Thematic analysis using NVIVO
Are the Women of Indian Sundarbans Living in the Dark? An Intersectional Analysis of Eye Health Care Seeking Among the Elderly	Indian Sundarbans, eastern Indian state of West Bengal	Mixed methods	Survey (*N* = 422)	Visually impaired elderly (174 M/268 F)	Quantitative data were analysed using STATA 11 and qualitative data analysed thematically using NVIVO 10
			In-depth interviews (*N* = 24)	Visually impaired elderly men and women (12 M/12 F)	
Gender Analysis of Family Care for Elderly: Evidence from Beijing, China	Beijing, China	Cross-sectional mixed methods study	Survey (*N* = 924)	Elderly (458 M, 466 F)	Analysed using SPSS 19, using Mann–Whitney U test; descriptive analysis; binary logistic regression
			In-depth interviews (*N* = 18)	Households with elders and one of their children (9 M/9 F)	
Strengthening Male Involvement in Prevention of Mother-to-Child Transmission of HIV in Enugu State, Nigeria	Enugu state, Nigeria	Qualitative	Document review	Journal articles, Research reports, Nigerian policy documents on national guidelines on PMTCT and integrated national guidelines for HIV prevention, treatment and care, Global reports on PMTCT strategic vision	Thematic content analysis using NVIVO 11
			In-depth interviews (*N* = 30)	Women and their male partners, *N* = 18 (9 F, 9 M) Health workers (doctors, nurses, pharmacists), *N* = 12 (4 M/8 F)	
			FGDs (*N* = 4)	Support groups (2 F, 2 M) FGD participants = 30, FGD F Enugu = 8, FGD F Nsukka = 8, FGD M Enugu = 6, FGD M Nsukka = 8	
Financing and Governance					
Male Involvement in the National Health Insurance Fund/Kreditanstalt für Wiederaufbau Prepaid Insurance Card for Pregnant Women in Pangani District, Tanzania	Pangani district,Tanzania	Qualitative	In-depth interviews (*N* = 6)	Male partners who re-enrolled/did not re-enroll	Thematic analysis
			FGDs (*N* = 5)	Female partners who re-enrolled/did not re-enroll, *N* = 3 (31 participants) Community health workers, *N* = 2 (4 M/7 F)	
			Group discussions (*N* = 8)	Healthcare providers, *N* = 5 (4 with female nurses and 1 with female nurses and one male nurse) (10 F, 1 M) Managerial teams, *N* = 3), (7F, 5 M)	
Mainstreaming Gender into PMTCT Guidelines in Tanzania	TANZANIA	Qualitative	Policy/ document review	Five PMTCT policy/strategy documents	Content analysis Thematic analysis
			Key informant interviews	Leaders of the health facilities and heads of reproductive and child health units involved in PMTCT (*N* = 26)	

*FGD* focus group discussion, *F* female, *M* male, *PMTCT* prevention of mother-to-child transmission of HIV/AIDS

Some areas of health systems are more commonly understood to be influenced and affected by gendered norms and social relations than others. For example, most people believe that services need to take account of the sex of the service user because the types of ill-health that they experience may be different. Similarly, there is little disagreement that men and women have different levels of access to health services. However, when it comes to issues like human resources for health, financing and governance, it is often assumed that these realms are gender neutral or unmarked by gendered concerns or power relationships. However, in practice, they are deeply influenced by gender – as is reflected in the studies included within this paper.

Within the health system, gender power relations affect, for example, the health workforce (whether informal care provided at home is recognised and supported; whether recruitment, retention, promotion and harassment policies take gender bias into consideration), health financing (the extent of financial protection availability to different groups, out-of-pocket expenditures) and governance (the systems of daily management, leadership, accountability and the extent to which policies incorporate gender considerations) [1, 4, 11–14].

This paper begins with an overview of the methodologies and methods used across the nine studies, followed by an exploration of key themes that emerged in terms of the application of a gendered approach to health systems research. The paper concludes with a discussion on the importance of incorporating gender analysis into health systems research and promoting this important agenda.

## Methods

The research used a combination of mixed, quantitative, qualitative and participatory methods, demonstrating the applicability of diverse research methods for gender and intersectional analysis. Within each study, the researchers adapted and applied a variety of gender and intersectional tools to assist with data collection and analysis, including different gender frameworks. Some researchers used participatory tools, such as photovoice and life histories, to prompt deeper and more personal reflections on gender norms from respondents. Others used conventional qualitative methods (in-depth interviews, focus group discussion (FGDs)). Care was also taken to think about multiple power relations that guide interactions within the particular communities of interest throughout the research process, for example, within sampling techniques (i.e. who is included and excluded from the research process), the positionality of the researcher and how this affects the data collection process, separating different categories of people during FGDs (i.e. conducting separate FGDs with older and younger women or with health managers and community health workers), and considering where participants, such as health workers and community members, would feel most comfortable for interviews (e.g. health workers at health facility and community members at community offices like village offices), as well as bringing gender and intersectionality lenses to the analysis process (e.g. exploring how gender, age and location shape progression opportunities within the realities of post-conflict contexts).

An overview of the methodology, methods and participants of each study is provided in Table 1.

To facilitate data collection and analysis, RinGs supported processes of joint reflection among the principal investigators of these research projects that enabled the examination of theoretical literature and empirical findings on the ways in which gender and other axes of inequity shape experiences across the health systems building blocks [15] and explore the strengths, weaknesses and challenges faced in undertaking gender and intersectional analysis in health systems research. This took the form of face-to-face interactions at a workshop in Kilifi, Kenya, in 2016; conference sessions and presentations; a webinar on how to conduct gender analysis; a webinar where grantees presented their research findings; evaluation processes (i.e. online surveys); contributions to blogs and case study reader; and joint analysis through email and additional face-to-face interactions at conferences and project meetings. Through this process the authors gained an in-depth awareness of the findings across the nine studies. These findings were reviewed and five core themes that cut across the different projects were identified and summarised, namely the intersection of gender with other social stratifiers; the importance of male involvement; the influence of gendered social norms on health system structures and processes; reliance on (often female) unpaid carers within the health system; and the role of gender within policy and practice, which have been used to structure the results section below. Each author was given the opportunity to review the core themes and provide input in relation to their individual studies or their awareness of the other studies.

Reflexivity is a central tenant of gender and intersectionality research and we recognise that our positionality will influence interpretation of the individual study findings and the core themes identified across the studies. The authors come from diverse backgrounds and locations. The RinGs Consortium Steering Committee members are from both high-income and low- and middle-income countries, and the study contributors are from low- and middle-income countries. Each study contributor was a national and resident of the country in which the study was conducted. Authors have varied sexes, ages, and ethnic and national backgrounds, as well as different types and levels of graduate training. For many of the study contributors, this was the first time they had used gender analysis as a lens within health system research, while others were more experienced. All study contributors were supported by the RinGs Steering Committee throughout the data collection and analysis process, the members of which have extensive experience in using gender analysis within health and health systems research. We feel that the diversity of locations and identities of the authors helped to provide a more robust analysis across the nine studies.

## Results

### The intersection of gender with other social stratifiers

Demographic changes have resulted in rapidly aging populations and/or increasingly urban populations in many settings and national systems are being called upon to respond to the health needs of people on the move (internally and internationally) [16, 17]. These demographic changes are also influencing the types of diseases (non-communicable diseases and re-emerging zoonoses) that health systems have to deal with. Risk factors for these diseases are highly influenced by social stratifiers, and thus different for people of different age, class, gender, ethnic identity, etc. However, few health systems research studies have paid explicit attention to the interaction of social stratifiers such as, for example, the ways in which gender, age, location, or migrant status shape the types of services that should be offered and how these services could be made more accessible [7]; this is particularly important for those interested in building ‘future health systems’ [4]. By conducting an intersectional analysis, the issue of age gained prominence in the India case study, while migrant status was shown to be an important factor in western Uganda.

In the Indian Sunderbans, we found that perceived poverty status was inversely associated to eye health status in the case of elderly women when compared to elderly men. Overall, within the four sample groups – poor female, non-poor female, poor-male and non-poor male, slightly more than half of poor females had low vision (52%), which was much higher than poor males (38%). Compared to non-poor males (23%), blindness was more prevalent among non-poor females (32%). Financially better-off older women were more likely to suffer visual impairments and blindness than their poor male counterparts (relative risk ratio (RRR) for low vision 0.36, confidence interval (CI) 018–0.70; RRR for blindness 0.48, CI 0.31–0.64). When gender was considered alongside education, level of education was found to have a significant association with level of visual impairment among men only. For example, both educated men and women were less likely to develop blindness, however, the result is significant only in the case of educated males (RRR for low vision 0.39, CI 0.35–0.44; RRR for blindness 0.22, CI 0.20–0.24). Without applying an intersectionality lens to the analysis, these distinctions would be lost. This has implications for health research, as research which only considers a single stratifier will miss different degrees of vulnerability across these categories and may offer recommendations which do not adequately meet the needs of those most vulnerable.

The relevance of social stratifiers varies by context. In the Ugandan study among migrant populations, different stratifiers, such as ethnicity and migrant status, were shown to be relevant. Migrants struggled with financial constraints and social norms that hindered access to maternal and newborn services, but importantly, they also reported negative experiences with healthcare workers brought on by ethnic discrimination (including extortion for additional informal payments), which meant that they did not take up services that should have been ‘available’ to them. Respondents explained that, when they reached health facilities, health workers identified their ethnicity by their names or their ability to speak the local dialect. Sometimes a simple greeting in the local dialect is used as a ‘screening tool’ to separate indigenous from migrant populations:

“*For example, I am a Lugbara, another mother is an Alur and then a Munyoro mother. If a nurse came and asked us in Munyoro only one mother will reply, those who do not answer back in the language will be abandoned, the Munyoro mother will be attended to first. At that moment, you will automatically know that she was picked because of tribe, you become timid, they will even start barking at you because you cannot explain your problems, even when nurses talk, you don’t understand.*” (Respondent 1, FGD 1 women group)

These studies emphasise that there is a need for analysing how gender intersects with other social identifiers, such as age and ethnic/migrant status, to influence health outcomes, access to services and experience at health facilities, and also how the salience of each particular stratifier depends on the context and should not be assumed a priori.

### Importance of male involvement

Research in gender can easily be skewed to focus only on the target study population, without including the ‘invisible gatekeepers’ or decision-makers who shape the contexts in which the studied power relations or gender inequities happen. As shown in the discussion which follows, research processes need to engage and triangulate data from gatekeepers or decision-makers, such as men and women in power, without further disempowering disadvantaged women or other marginalised people. The importance of taking these gendered power relations into account emerged as a significant factor in relation to women’s access to health services and progression within the health sector in the studies from Cambodia, Nigeria and Tanzania.

In Cambodia, in all political periods, women working in the health system needed support from men, both at the household and institutional levels, to facilitate their participation, progression and leadership within the health system. For example, in the 1980s and 1990s when health workers were required to retreat with soldiers in the remote conflict-affected areas to provide healthcare services, six female managers claimed the importance of having the support of their male peers, which allowed them to stay on duty. In all periods after conflict (from 2000s to present day), male partners respecting women’s decisions and taking responsibility for domestic work (such as child care) to support women’s professional development, and institutional support from male leaders, were vital to women’s career progression. All female facility managers, for example, acknowledged the importance of strong support from the (male) head of the local institution in providing advice, particularly in the early stage of their leadership role:

“*…I never expected a man, being a leader, valued women like this because generally a man is likely to get promoted. However, my chief was different from others; he promoted a woman.*” (Married health manager, female)

In contrast, the lack of male involvement was considered a key challenge in the uptake of prevention of mother-to-child transmission (PMTCT) services offered to women in Nigeria. The research explored the extent of male involvement in PMTCT and its effects on women’s access to and use of PMTCT services. Findings showed that only a few men accompanied their wives to antenatal care and men’s attendance at educational sessions on prevention of unintended pregnancies and HIV counselling and testing was low. Factors which contributed to the limited participation of men in PMTCT occurred at the individual, community and the health system levels. In terms of individual and relationship factors, time constraints, due to opportunity costs in terms of loses that will be incurred in some households if men are away from their private sources of income-generating activities, poor spousal communication, and non-disclosure of status to one’s partner came to the fore. At the community level, gendered norms and expectations, such as pregnancy being perceived as women’s responsibility, male dominance in household decision-making, which makes some men feel that if they accompany their wives to PMTCT services they are being controlled by them, and ridiculing men who accompanied women to antenatal care visits were common. At the health system level, women-centered antenatal care services, unwelcoming attitudes of health workers to men who accompanied their partners, cost of transportation to access antiretroviral treatment (ART) in distant health facilities in order to avoid stigma, and the fact that appointments for ART for partners were scheduled separately, which contributed to couples attending ART clinics individually, affected PMTCT uptake.

Community level: “*…What happens to men all the time is that they feel that family planning is always for the women… that it is the women that need such services…that is why I don’t follow my wife to go for it.*” (P6, FGD male support group, Nsukka)

Health system level: “*The attitude of the nurses at times contributes a lot to why some* [men] *don’t like to follow their wives for antenatal.*” (P2, FGD Male support group Enugu)

Overall, the lack of men’s involvement in PMTCT was found to contribute to difficulty in breastfeeding properly or as recommended for HIV-positive women, difficulty with keeping scheduled antenatal care appointments, inability to discuss outcomes of counselling visits, and prevention of unwanted pregnancies and poor adherence to antiretroviral treatment. Several benefits of male participation were identified and included male partners increased understanding of and accepting health programmes, women being freer to access programmes, reduction of the effects of male dominance on access to and uptake of PMTCT, male partners being better positioned to support their female partners emotionally and financially, couples being more able to work together towards preventing unintended pregnancies, and the promotion of adherence to treatment.

The study in Tanzania explored male involvement in the National Health Insurance Fund/Kreditanstalt für Wiederaufbau^1^ prepaid insurance card for pregnant women, a programme aimed at improving maternal and child health through the provision of insurance coverage to poor families. Maternal mortality is found to be linked to a wide range of factors in women’s lives, including lack of access to timely emergency obstetric care, the value placed by women, their families and communities on women’s health, women’s economic position, their access to education and information, and women’s capacity to make autonomous decisions [18]. Lack of male involvement in planning or implementing interventions has also been found to be a major barrier to achieving improved maternal and child health [19].

This study explored the factors associated with male involvement or lack thereof in the programme and identified strategies that can be used to increase it. It found that programme implementation, cost, health system challenges, and social norms act as facilitators and barriers in terms of male partner involvement in the implementation of the National Health Insurance Fund/Kreditanstalt für Wiederaufbau insurance programme. Despite it not being the norm in Tanzania, this programme encouraged men to accompany women to healthcare centres during antenatal care visits to ensure that women and their partners were tested together for HIV and educated on how to take care of the pregnancy and prepare for delivery. Because men were encouraged to accompany their partners to the health facility, they became aware that the programme paid for services that their wives received and that no further financial contributions would be required from either the woman or her male partner. This positively affected male support for receiving care:

“*Nowadays he has changed, when you fall sick he asks you to go to the facility as he knows it’s free...he cannot say I don’t have to go because we do not have money... if I wouldn’t have gone with him the first day he could not know...*” (FGD – women at Pangani Hospital)

What these Cambodian, Nigerian and Tanzanian studies demonstrate is the need for male involvement and support across the health system, including within human resources for health, access to healthcare and service delivery. If gender equitable health systems are to be achieved, male gatekeepers or decision-makers who influence the contexts in which poor women live and work need to be involved, alongside women who have to be able to make (and finance) autonomous choices about their health (see [20]). Health systems actors should support the equitable distribution of domestic work and discriminatory systems in which women are reliant on male patronage must be reformed. Future studies should explore how gender power relations affect men’s health system needs, experiences and outcomes. In addition, research must examine how male involvement can be part of a programme to improve gender equity more broadly to avoid the potentially harmful impacts if initiatives reinforce patriarchal power.

### Gendered social norms influence health system structures and processes

The studies in Zimbabwe and Uganda exploring human resources for health both showed how gendered norms shaped the types of employment men and women had within the health system, and what roles were considered feasible and acceptable. Within Zimbabwe, for example, participants not only reported that the health sector was largely feminised, with women accounting for the majority of healthcare workers, but also that there were gender imbalances within the different professions in Zimbabwe, stemming from the careers men and women tended to pursue and the training recruitment processes.

The Zimbabwean case study also found that access to training opportunities and career development were shaped by gender roles and norms at the household and institutional levels. Men were able to take up training opportunities, some of which were self-funded, whereas women were often unable to pursue these opportunities due to gendered family responsibilities and a lack of personal financial resources. In terms of relocation for career development or new opportunities, women were much more likely to follow their husbands. Here, gender intersected with marital status in a way that was advantageous for men but disadvantageous for women. This meant that female health workers often had to resign from their jobs to seek new ones, sometimes in a different sector (such as moving from the government to the mission sector), sacrificing the accruing of years of service required to access training and the opportunities for promotion. This affected their career levels; many reported re-joining the health sector in junior/lower posts, and therefore a loss of pay/accepting lower pay, delaying their time for promotion, upgrading and/or upskilling.

“*It* [my husband’s relocation] *affected me because when I went for upgrading, other upgradings were already done and I was told that my name was once listed at my previous posting location and it was said that ‘No this one resigned from this hospital so she will find other things where she is’, secondly most of my juniors are now Sisters-in-Charge, they always laugh at me that they have been promoted before me, so it affected me so much. I think if I was still there I was going to be one of the seniors there.*” (Married female health worker)

In Uganda, although responsibilities were the same for both male and female community health workers (CHWs), CHWs reported that, in practice, they were involved in different types of work depending on their gender. Female CHWs were more likely to be available in communities to address local problems, and more involved in child care. In this context, due to their privileged ownership and access to motor vehicles, male CHWs were able to assist patients with referrals to facilities during health emergencies, cover larger geographic distances during community mobilisation activities and take up supervisory responsibilities. The gendered division of labour within communities also meant that male CHWs were more involved in manual work such as supporting renovation of latrines and cleaning wells.

“*As a male CHW, I can use any means of transport available like a bicycle or motorcycle or car to transport a patient to a health facility in case of any problem in the community. For a female CHW, it might be hard to use certain means of transport like bicycles to transport a patient to the hospital*.” (Male CHW, Photographer 10, age 31)

Echoing the implicit hierarchy shown to exist in Uganda, in the Cambodian research study, women in leadership positions felt that male leaders were more valued. For example, female managers felt that their voice was less respected; being younger women and having a lower level of technical skill compared with men was also seen to influence respect received. Here, gender intersected with educational level, professional cadre and age to influence experience within the health system.

“[In the meeting] *mostly they* [senior health system staff] *accept men’s ideas because they believe that men have a long-sighted vision. Moreover, men are more well-educated than me; most of them are doctors and I am just a medical associate. Therefore, my skill and knowledge are lower than them…*” (Married, health manager, female)

“*Some people are disappointed* [with me] *as he is older* [than me] *and I became his chief.*” (Single, health manager, female)

These studies demonstrate the gendered experience of health workers, reflecting strategic positioning, gendered access to resources, gendered division of labour and gendered social norms, as well as how these influence occupational roles and career development within the health system.

### Reliance on (often female) unpaid carers within the health system

Two of our studies pay attention to the issue of unpaid care, which forms the foundation of the health system and which is normally performed by women in the household and the community. The study in Wakiso District, Uganda, focused on CHWs, a cadre of worker which is often simultaneously lauded as a crucial force in expanding health service access whilst being notoriously underpaid (or not paid at all) and under-supported by the health system [21]. The Ugandan Ministry of Health introduced CHWs, locally referred to as Village Health Teams, in 2001, to mobilise individuals and households for better health [22, 23]. In Uganda, the majority of CHWs are unpaid female community volunteers who can read and write (preferably in their local language), are selected by local leaders, and trained by health professionals to provide accurate health information and referral. CHWs carry out health education, conduct household visits to promote sanitation and hygiene, mobilise the community for public health interventions such as immunisation, treat children below 5 years of age under integrated community case management of childhood illnesses, and refer patients to health facilities.

In China, unpaid healthcare is undertaken by family members who look after elderly relatives; 98% of the elderly rely on daily care provided by family members, and only 2% rely on care from health professionals and social welfare organisations. Due to the lack of well-functioning, long-term care programmes, as well as the historical filial piety tradition (the belief that adult children have the responsibility to support their parents), Chinese elderly prefer to rely on family members (usually daughters or daughters-in-law) in their final years of life [24].

Our Chinese study found that, while a traditional support system was still mainstream in rural areas, transition was underway in urban areas. For example, all (18) of the rural elders interviewed were cohabiting with their children while only one of the urban elders was cohabiting with offspring. In rural areas, the tradition of “*bringing up sons to support parents in their old age*” was still accepted and sons and daughters-in-law were the core force in supporting parents. In urban areas, all but one of the elders interviewed thought that daughters were more reliable than sons, and daughters provided more hours of care compared to sons and daughters-in-law. Overall, the study concluded that unpaid elderly care is an important part of the health system, particularly in rural areas, and better understanding of who provides and receives care is important in terms of identifying weaknesses and gaps in provision and responding with social policy. Due to China’s transition from a traditional filial piety system, social welfare systems such as long-term care insurance and professional care institutions need to be strengthened if the elderly are to receive the care they need. The studies reported in this section show how intensely health systems rely on unpaid – and often unacknowledged – care.

### Gender in policy and practice

Health systems policy development does not always pay adequate attention to gender and, even when these policies include gender, good intentions can ‘evaporate’ when it comes to measurable indicators and actual implementation [2, 25–27]. In addition, policy-makers often have limited capacity or knowledge about gender and gender inequities, which limits its recognition and inclusion within policy and practice. Gender relations and roles need to be considered when designing and implementing programmes within the health system to ensure that health systems serve to address gender inequalities and advance health outcomes equitably [2, 28, 29].

Our Tanzanian study analysed policies for the prevention of mother-to-child transmission of HIV [14]. Mainstreaming gender into PMTCT programming is expected to lead to increased coverage, efficiency and effectiveness of services, contributing towards a reduction of child and maternal illness and mortality [30]. Using a gender framework, this study assessed whether relevant Tanzanian guidelines and policies were gender unequal, gender blind, gender sensitive, gender specific, or gender transformative.

While gender-related issues were mentioned in all of the guidelines explored, indicating some degree of gender responsiveness, the policies were found to fall short of the gender-transformative ideal. For example, the policy documents recognised gender inequality in decision-making and access to resources as a barrier to accessing PMTCT services by women; however, no attempt was made to transform harmful gender norms, roles or relations. Actions to transform masculinity norms that discourage men from seeking care, taking an HIV test, or accompanying their partners to PMTCT clinics were not included. This might be explained by how gender is often handled as an ‘add on’ in order to fulfil certain requirements, rather than being made an integral part of the entire policy. Overall, this Tanzanian study concluded that revision of guidelines to mainstream gender is greatly needed if PMTCT services are to effectively contribute towards a reduction of child and maternal morbidity and mortality. All of the studies above demonstrate the need for policy and practice responses to consider gender if inequitable systems and structures within the health system are to be transformed.

## Discussion/Conclusion

Health systems research has long sought to address gender inequities in health with little success [4]. There is a growing body of theoretical literature on the importance of using a gender lens within health systems research [1, 2, 31]. However, there are few illustrations of how to put this approach into practice or how to extrapolate from individual studies to benefit health systems research.

The nine studies presented here offer new insights into the role of gender analysis within health systems research. First, in relation to using a gender and intersectional approach, the studies demonstrate the need to pay attention to a wide variety of social stratifiers that influence women’s access to health services. The examples included are age, education and gender in the Indian Sundarbans in relation to eye health, and migration, gender and ethnic identity in relation to maternal health in Uganda. This research, which identifies particular stratifiers and details how these intersections shape particular women’s experiences, is relatively unique as the application of intersectionality to health systems research is still in its infancy [7]. Intersectional approaches are attractive for their ability to go beyond the binary categories of male and female, to offer new forms of disaggregation of data, and for their emphasis on transformational change [32]. The findings presented here suggest that more research needs to explore the diversity of factors – generally considered to lie outside the health system – which shape how people respond to the health system and how the health system responds to them.

Secondly, the application of a gender lens demonstrates the importance of addressing the power relations underlying men’s involvement in health programmes directed towards women, drawing on examples from Cambodia, Tanzania and Nigeria. In Tanzania, it was shown that the provision of additional financial resources bolstered men’s support for their wives’ use of maternal and child health services. Looking across these studies, an intersectional approach suggests that more attention now needs to be paid to understand men’s positions and vulnerabilities, the potential benefits or harms caused by their involvement, as well as to understand why their support is lacking. Questions that need further exploration include, what are the current ways in which social stratifiers intersect to marginalise men? What are the potential harms that stem from men’s involvement in maternal and child health programmes? How do gender power relations affect men’s health system needs, experiences and outcomes? What power relations and institutional arrangements bolster and reinforce men’s right to make decisions over the bodies and health of women and children?

Thirdly, these studies demonstrate the importance of gender in relation to health workers (both paid and unpaid). Examples from Zimbabwe and Uganda show that gender norms and roles mean that male health workers are better able to use the system to advance their careers but also that, in the case of Uganda, this leads to a heavier physical burden on men. Moreover, men’s support, both as family members and as health system employees, is vital for women who seek to achieve positions of leadership within the health workforce. The implications of these gendered processes in health system service delivery need further exploration (see [33, 34] for work in this area).

Finally, using a gender lens directs attention to the policy arena and the ways in which policy can itself be limiting, despite aspirations to support gender-positive transformation. A detailed study of Tanzania’s PMTCT services is provided above, which shows the need to go beyond the inclusion of gender in policy to seek to promote gender-transformative approaches [14]. Other studies supported by our initiative also demonstrate that moving from policy to implementation reveals further gender dynamics that are at times unanticipated. The implications of the diverse examples of gender and health systems research highlighted indicate that policy-makers, health practitioners and others interested in enhancing health system research and delivery have solid grounds to advance their enquiry and that one-size-fits-all heath interventions that ignore gender and intersectionality dimensions require caution. It is essential that we build upon these insights in our efforts and commitment to move towards greater equity both locally and globally.

### Strengths and limitations of the study

This analysis brings together a wide group of scholars focusing on different aspects of gender, intersectionality and health systems research. Each study explicitly utilised a gender lens throughout the formulation and implementation of the research. Due to word restrictions we are unable to provide robust detail about each study’s methodology. We are unable to claim generalisability of this analysis due to the diverse research methods used, however, we believe the core themes identified will be transferable to many low- and middle-income country contexts. While gender power relations are highly context specific, the fact that we were able to identify core themes across nine studies conducted in diverse contexts demonstrates the permeability and perviousness of gender inequities globally.

## Abbreviations

ART: anti-retroviral treatment
CHW: community health workers
FGD: focus group discussion
PMTCT: prevention of mother-to-child transmission of HIV/AIDS
RinGs: Research in Gender and Ethics: Building Stronger Health Systems Consortium
RRR: relative risk ratio

## Footnotes

1 Kreditanstalt für Wiederaufbau, which is German Development Bank, one of the funders of the programme.
